# Social amoebae trap and kill bacteria by casting DNA nets

**DOI:** 10.1038/ncomms10938

**Published:** 2016-03-01

**Authors:** Xuezhi Zhang, Olga Zhuchenko, Adam Kuspa, Thierry Soldati

**Affiliations:** 1 Department of Biochemistry, Science II, University of Geneva, Geneva 1211, Switzerland; 2 Verna and Marrs McLean Department of Biochemistry and Molecular Biology, Baylor College of Medicine, One Baylor Plaza, Houston, Texas 77030-3498, USA

## Abstract

Extracellular traps (ETs) from neutrophils are reticulated nets of DNA decorated with anti-microbial granules, and are capable of trapping and killing extracellular pathogens. Various phagocytes of mammals and invertebrates produce ETs, however, the evolutionary history of this DNA-based host defence strategy is unclear. Here we report that Sentinel (S) cells of the multicellular slug stage of the social amoeba *Dictyostelium discoideum* produce ETs upon stimulation with bacteria or lipopolysaccharide in a reactive oxygen species-dependent manner. The production of ETs by S cells requires a Toll/Interleukin-1 receptor domain-containing protein TirA and reactive oxygen species-generating NADPH oxidases. Disruption of these genes results in decreased clearance of bacterial infections. Our results demonstrate that *D. discoideum* is a powerful model organism to study the evolution and conservation of mechanisms of cell-intrinsic immunity, and suggest that the origin of DNA-based ETs as an innate immune defence predates the emergence of metazoans.

## Abstract (web summary)

Neutrophils secrete net-like structures made of DNA and anti-microbial peptides, which can trap and kill extracellular pathogens. Here, the authors show that such nets are also produced by so-called Sentinel cells in the multicellular slug stage of the social amoeba *Dictyostelium discoideum*.

## Graphical abstract

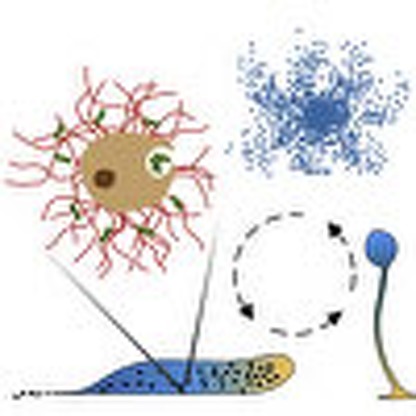

The circulating phagocytes of the human innate immune system represent an ancient anti-microbial host defence. In addition to direct microbe engulfment and secretion of anti-microbial agents, activated phagocytic neutrophils also eliminate microbes by releasing extracellular traps (ETs)^1^. ETs produced by neutrophils were originally described as reticulated nets of DNA decorated with anti-microbial granules that contribute to innate immune defense by capturing and killing extracellular pathogens^1^,^2^. Since these pioneering studies, other innate immune phagocytes have also been shown to produce ETs^3^, and the specific type of programmed cell death often associated with ET generation has been coined ETosis^4^. Recent evidence strengthen the emerging concept that pathogens that are too large to be phagocytosed, such as fungal hyphae and bacterial aggregates, trigger ETosis^5^,^6^. Among other stimuli, bacterial lipopolysaccharide (LPS) is able to activate neutrophils to produce ETs^1^. ETs found *in vivo* at sites of infection and inflammation are able to control the spread of microbes as well as to increase the local concentration of anti-microbial proteins around the trapped microbes^7^. The process of ET formation is tightly regulated by the reactive oxygen species (ROS)-generating NADPH oxidase 2 (NOX2). Consequently, Chronic Granulomatous Disease (CGD) patients lacking functional NOX2 are not only deficient in producing an oxidative burst, but also in ETosis, thereby contributing to severe and recurrent bacterial and fungal infections^8^. Recent integrated findings indicate that ETosis is an ancient host–defense mechanism shared by several specific phagocytic cell types across vertebrates and invertebrates^3^,^9^,^10^ However, the evolutionary origin of this anti-microbial mechanism, and whether it is a feature unique to the animal kingdom is still unclear.

Phagocytosis, the core function of the innate immune response, is highly conserved between human professional phagocytic cells and amoebae, a sister group to the animals and fungi that branched after the divergence of plants^11^,^12^,^13^. Therefore, the genetically tractable free-living amoebae *D. discoideum* provides a unique system to study the evolution and conservation of the mechanisms of innate immunity. In their natural habitat, solitary *D. discoideum* cells feed on soil bacteria, while the laboratory strains can be cultured in axenic medium, and easily genetically modified. As illustrated in Fig. 1a, food depletion induces a remarkable developmental program, in which around 100,000 amoebae aggregate to form a migrating multicellular slug. This stage can be maintained up to 48 h under laboratory conditions, until it eventually undergoes terminal differentiation and culmination into a fruiting body comprised of the spore mass supported by a cellular stalk^14^. The slug consists of a few specialized cell types; however, the Sentinel (S) cells are the only cell type with phagocytic capacity, thus functioning as a primitive innate immune system^15^. S cells are continuously produced within the slug to phagocytose invading microbes, and then are sloughed off as the slug migrates. By gentle physical treatment, the sampled slugs can be disaggregated into small cell clusters and even single cells, allowing targeted analysis of specific cell types after purification.

In this study, we use the social amoeba *D. discoideum* as model organism to examine whether a DNA-based bactericidal strategy already functioned as a primitive innate defense system long before the emergence of animals. Here we report that in the multicellular slug stage of *D. discoideum*, only the S cells produce DNA-based ETs in a ROS-dependent manner for clearing bacteria. *D. discoideum* Toll/Interleukin-1 receptor domain-containing protein TirA and NOX enzymes that are conserved between human and *D. discoideum* play a crucial role in the production of ETs. Our results demonstrate that *D. discoideum* is an excellent model organism to study the functional genetics and the evolution of innate immunity.

## Results

### Production of ETs from *D. discoideum* S cells

**Figure 1:**
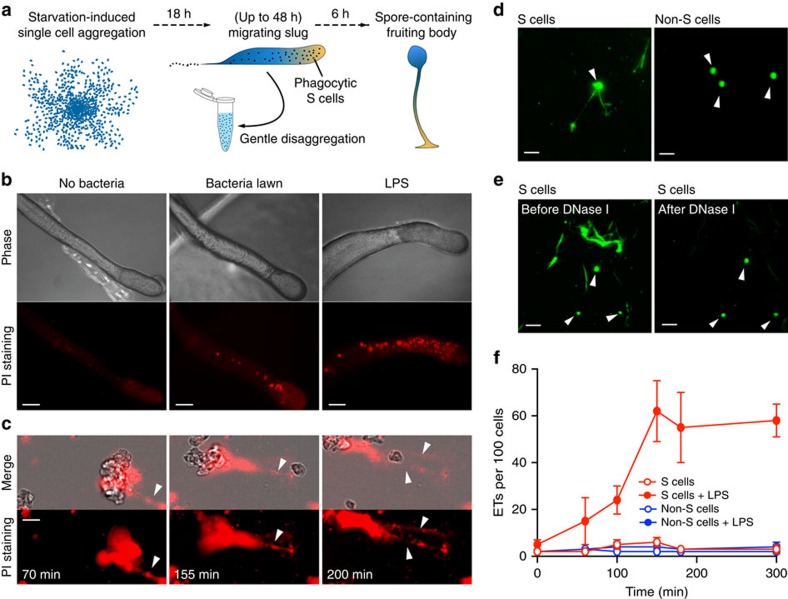
Bacteria or LPS stimulate S cells to produce ETs. (**a**) Development of *D. discoideum* from single cells to a multicellular organism. The black dots in the migrating slug indicate S cells. (**b**) Red fluorescent dots (indicative of extracellular DNA) from AX4 slugs migrating on PI-containing agar plates in absence or presence of *K.p.* or *K.p.* LPS. (**c**) Disaggregated AX4 slug cells release ETs (arrowheads, PI staining) on LPS stimulation. See also Supplementary Movie 1. (**d**) On LPS stimulation, DNA fibres (arrowheads, SYTO9 staining) were released from FACS-purified S cells, but not from non-S cells. (**e**) ETs disappeared after 10-min exposure to DNase I, while the protected nuclear DNA remains visible (arrowheads). (**f**) Quantification of ETs production from purified S and non-S cells with or without LPS exposure, *n*=3. Error bars, s.e.m. Scale bars, (**b**) 100 μm; (**c**–**e**) 10 μm.

To test the hypothesis that S cells employ ETs for bacteria clearance, we starved amoebae to generate migrating slugs on agar plates containing propidium iodide (PI), a membrane-impermeant dye that stains extracellular DNA. When slugs migrated onto a lawn of *Klebsiella pneumoniae* (*K.p.*) or an area containing LPS, the number and size of fluorescent red punctae, indicative of extracellular DNA release, increased dramatically compared with the ones without bacteria or LPS exposure (Fig. 1b). After disaggregation, we also observed that some slug cells exposed to LPS released filamentous structures that became red fluorescent in the presence of PI (Fig. 1c and Supplementary Movie 1). These PI-positive structures are morphologically different from the compact staining of the nuclei of the few dead cells and are thus easily distinguishable. In fact, these structures were very similar to the ETs produced by activated human neutrophils^1^. We then isolated S cells and non-S cells by fluorescence-activated cell sorting^15^, and monitored ET production after LPS stimulation with the cell-permeant DNA dye SYTO9. S cells clearly produced ETs while non-S cells did not (Fig. 1d), and the addition of DNase I quickly digested the extracellular fibres (Fig. 1e). Stimulation of S cells with LPS triggered a 10- to 20-fold increase in ET production (Fig. 1f). The small number of ETs observed with non-S cells was likely due to a contamination with S cells (Fig. 1f). Stimulation with *K.p.* bacteria also induced ET production only in S cells. These experiments indicate that, as in mammals, the innate immune cells of the *D. discoideum* slugs are the only cells capable of elaborating DNA-based ETs.

### Trapping and killing activities of ETs

**Figure 2:**
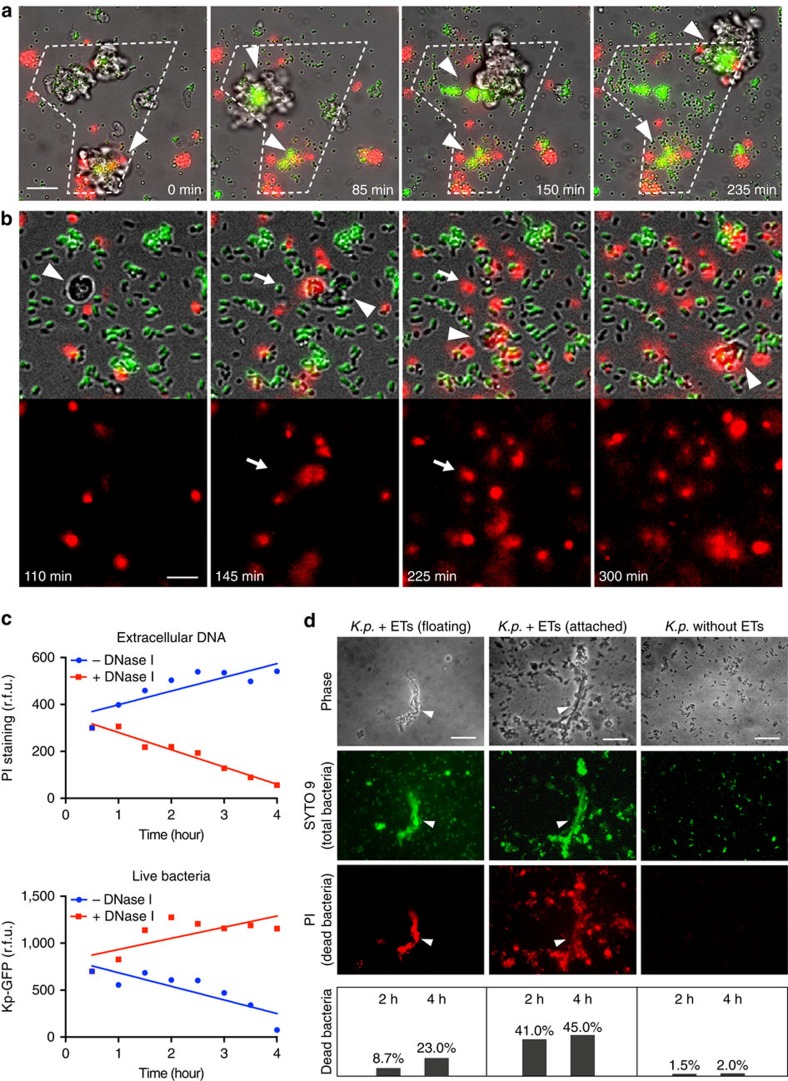
DNA-based ETs trap and kill extracellular bacteria. (**a**) The dashed line outlines the zone explored by migrating disaggregated slug cells during the time of the experiment. The floating green fluorescent latex beads were progressively trapped at the centre of cell clusters and on their migration tracks (arrowheads) in the presence of PI (red), where extracellular DNA becomes concentrated. See also Supplementary Movie 2 and Supplementary Fig. 1. (**b**) Migrating S cells (arrowhead) from disaggregated slugs not only kill GFP-expressing *K.p.* by phagocytosis, but also kill them extracellularly during migration, as revealed by the sudden loss of green fluorescence and PI penetration (arrow). See also Supplementary Movie 3 and Supplementary Fig. 2. (**c**) Quantification of **b** in a microplate reader shows a representative result from three independent experiments. Compared with controls, addition of DNase I decreased extracellular DNA (PI staining), and increased bacteria survival (*K.p.* GFP intensity). (**d**) Purified S cells from AX4 slugs were mixed with *K.p.* at a 5:1 ratio of bacteria to amoebae to stimulate ET production. The differential staining with PI and SYTO9 was used to measure the fraction of dead bacteria in suspension or attached to ETs. *K.p.* bacteria treated similarly but in absence of ETs (right) were used as a control. Arrowheads indicate ETs. The graphs present results from one representative of several experiments, counting hundreds of bacteria each time. Scale bars, (**b**) 10 μm; the rest 20 μm.

To analyse whether these extracellular DNA structures have trapping activities, we mixed disaggregated slug cells with 1 μm green fluorescent latex beads together with LPS and PI. As shown in Fig. 2a, Supplementary Fig. 1 and Supplementary Movie 2, while the extracellular DNA was released from S cells and cell clusters, the floating green beads gradually accumulated with extracellular DNA in the migration tracks of those cells, as well as at the centre of the cell clusters. The gradual increase of green fluorescence intensity within the marked region and the co-localization of green and red fluorescence clearly suggest that ETs produced by S cells have strong particle-trapping ability. Notably, the DNA-bead aggregates left on the migration tracks are reminiscent of circulating S cells that are sloughed off during slug migration after phagocytosing bacteria. To monitor the bacterial killing activity of ETs, live green fluorescent protein (GFP)-expressing *K.p.* (Kp–GFP) were mixed with disaggregated slug cells, and PI to label both ETs and dead bacteria. Migrating phagocytic S cells (arrowhead) efficiently engulfed bacteria, but extracellular bacteria also died in their vicinity (arrow), indicated by the sudden loss of GFP fluorescence and acquisition of PI labelling (Fig. 2b, Supplementary Fig. 2, Supplementary Movie 3). The PI-positive redness around S cells increased noticeably without apparent amoeba cell death. To test whether extracellular death of bacteria correlated with DNA release from S cells, in parallel, we quantified the fluorescence intensity of extracellular DNA (PI staining) and live Kp–GFP in a microplate reader. Addition of DNase I decreased PI fluorescence, but significantly increased bacteria viability (Fig. 2c). An alternative method by live/death staining of *K.p.* bacteria in the presence or absence of ETs also confirmed the trapping and killing activities of ETs (Fig. 2d). These results indicate that, in addition to their bactericidal phagocytic capacity, *D. discoideum* S cells also produce ETs to kill extracellular bacteria.

### DNA composition of ETs

**Figure 3:**
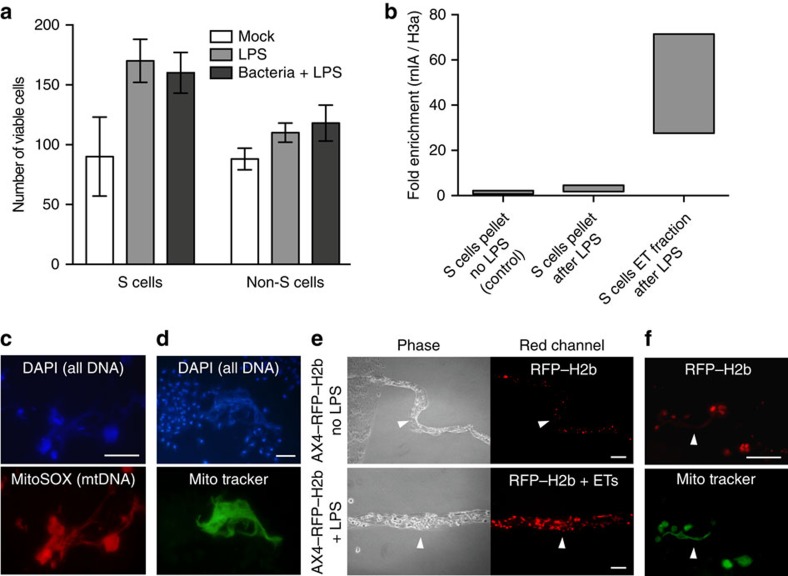
ETs are mainly composed of mitochondrial DNA. (**a**) The viability of purified S and non-S cells was not impaired after exposure to LPS or the combination of LPS and *K.p.*, *n*=3. (**b**) AX4 slugs developed from cells initially grown in axenic conditions were treated with LPS for ET stimulation followed by qPCR analysis. A representative result from three independent experiments performed in triplicates is shown. The range of relative folds of enrichment of mtDNA versus nuclear DNA compared with the control was calculated by the ΔΔCt method. (**c**,**d**) Visualization of ETs produced from disaggregated AX4 slug cells with MitoSox Red and MitoTracker Green staining after LPS stimulation (see Methods section ‘Visualization of ETs' for experimental details). The total DNA was visualized by DAPI staining after fixation. (**e**) Comparison of S cell-containing trails (arrowheads) left behind migrating slugs of AX4–RFP–H2b strain on PI-containing agar plates in the absence or presence of LPS. (**f**) ETs (arrowheads) produced by S cells from AX4–RFP–H2b slugs on LPS stimulation are positive for preloaded MitoTracker Green, but not for RFP–H2b. Error bars: s.e.m. Scale bars, (**e**) 100 μm; the rest 20 μm. qPCR, quantitative PCR.

Two types of ETs have been described in mammals. Production of nuclear DNA-based ETs accompanies ETosis^1^,^2^,^16^, whereas cells remain viable after production of mitochondrial DNA (mtDNA)-based ETs^9^,^17^. The migrating S cells do not appear to die during ET release (Supplementary Movie 3), and stimulation with LPS or bacteria did not compromise S cells viability (Fig. 3a). The increase in cell counts after incubation with LPS or bacteria likely reflects a decrease in cell clumping. Therefore it is very likely that the DNA of ETs produced by S cells is from mitochondria. To analyse the ET DNA composition, quantitative PCR on rnlA (mtDNA) and H3a (nuclear DNA) was performed on ET fractions and S cell pellets after LPS stimulation, with total DNA of non-stimulated S cells as a control. After LPS stimulation, the ratio of mitochondrial-to-nuclear DNA in the S cell pellet is not significantly different from the control. However, the ratio of mitochondrial-to-nuclear DNA in the supernatant of S cells stimulated by LPS showed a significant enrichment for mtDNA, ranging from 30- to 70-fold, compared with the control (Fig. 3b). In addition, the ETs produced from S cells can also be clearly visualized by MitoSOX Red and MitoTracker Green, which stain the mtDNA and mitochondrial proteins, respectively (Fig. 3c,d). Furthermore, in the trails of migrating slugs developed from AX4 strain expressing red fluorescent protein-labelled H2b (AX4–RFP–H2b), the red fluorescence was mainly compacted within the nuclei of S cells being sloughed off, and no obvious ET structure was stained on the PI-containing agar plate. However, when LPS was applied to the AX4–RFP–H2b slugs, the clouds of extracellular DNA surrounding the S cells were significantly stained by PI, indicating the presence of ETs (Fig. 3e). In addition, MitoTracker Green stains ETs produced from S cells of AX4–RFP–H2b, but the RFP–H2b signal is almost absent from these ETs (Fig. 3f). All these observations suggest that ETs produced by *D. discoideum* S cells are of mitochondrial origin and contain little or no nuclear chromatin, however, we cannot completely exclude that ETosis of S cells and nuclear DNA also contribute to ETs.

### Roles of ROS in ET formation

**Figure 4:**
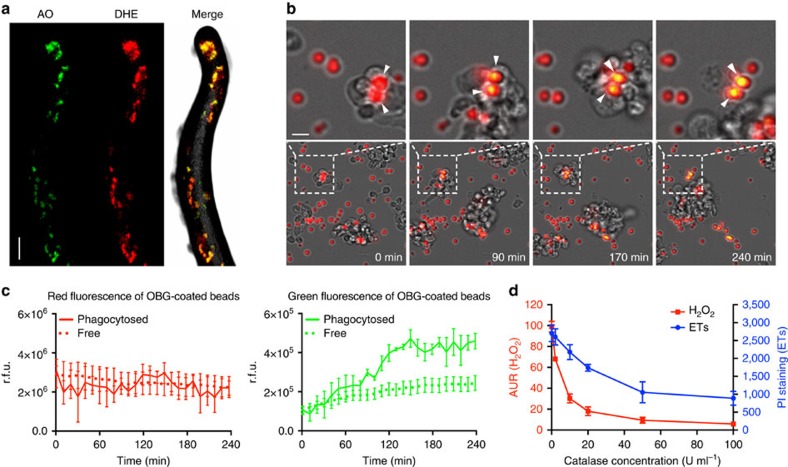
ET formation is ROS dependent. (**a**) ROS signals (DHE staining, red) and S cells (AO staining, green) co-localize in AX4 slugs stimulated with *K.p.* See also Supplementary Movie 4 and Supplementary Fig. 5. (**b**) The ROS-sensitive OxyBurst Green (OBG)-coated beads were phagocytosed and oxidized by S cells, indicated by the fluorescence shift of ingested beads from red to yellow (arrowheads). The dashed lines in the lower gallery indicate the regions showed at higher magnification in the upper gallery. (**c**) Quantification of the signals for three phagocytosed and three non-phagocytosed beads over time. (**d**) Addition of catalase dose dependently decreased the levels of H_2_O_2_ (AUR assay), as well as extracellular DNA released (PI staining) from LPS-stimulated AX4 slug cells, *n*=3. Error bars: s.e.m. Scale bars, (**a**) 100 μm; (**b**) 5 μm.

ROS are generated by aerobic organisms, and have served both killing and signalling purposes since early in evolution^18^,^19^. Activated human neutrophils generate large amounts of toxic ROS via the oxidative burst to kill phagocytosed microbes and to actively drive ET generation^2^,^5^. A previous study showed that the intracellular superoxide generation in *D. discoideum* can be stimulated by bacterial LPS, and detected by the ROS-sensitive dye dihydroethidium (DHE). The signals can be visualized and quantified in cell monolayers^20^. Among the various S cell-labelling dyes used in our pioneering study, the lysosomotropic dye acridine orange (AO) exhibited satisfying spectral compatibility with DHE^15^. To measure the *in situ* ROS production and visualize S cells in the slugs without causing structural damage, we gently sprayed DHE (red) and AO (green) onto slugs generated on a lawn of *K.p.*, respectively. In Fig. 4a, one of the sections of a confocal image stack is shown to illustrate the co-localization of the red and green fluorescent dots within the slug. The object-based three-dimensional (3D) reconstruction of the confocal images (Supplementary Movie 4) and pixel intensity spatial correlation analysis of eight independent slugs (Supplementary Fig. 3) showed high co-localization of red and green fluorescence, indicating that S cells are the main producers of ROS in the slug.

To visualize the process of ROS production by S cells, we fed the disaggregated slug cells with 3 μm silica beads coated with the ROS-sensitive dye OxyBurst Green (OBG) and the ROS-insensitive reference dye Alexa Fluor 594 (ref. 20). As illustrated in Fig. 4b and Supplementary Movie 5, the phagocytic S cells actively engulfed the beads, and the production of ROS by the S cells oxidized the OBG dye from non-fluorescent to green fluorescent, turning the beads to a bright yellow colour (arrowheads). Quantification of fluorescence intensity showed that the beads phagocytosed by S cells exhibited significantly higher oxidation levels than the non-phagocytosed beads, (Fig. 4c), suggesting that S cells can generate ROS in their phagosomes, as shown previously for vegetatively growing amoebae^20^. However, we cannot exclude that other slug cell types may also generate low amounts of ROS.

These results made it plausible that ROS are the signalling molecules that activate S cells to produce ETs. If this were correct, ROS scavengers should suppress ET formation. We applied Amplex UltraRed (AUR) and PI on LPS-stimulated slug cells to monitor extracellular H_2_O_2_ production^20^ and ET formation^1^,^21^, respectively. Addition of the H_2_O_2_-scavenging enzyme catalase decreased both the H_2_O_2_ concentration and ET formation in a dose-dependent manner (Fig. 4d), indicating that extracellular ROS lie upstream of and contribute to ETs formation.

### Genetic manipulation of ET generation

**Figure 5:**
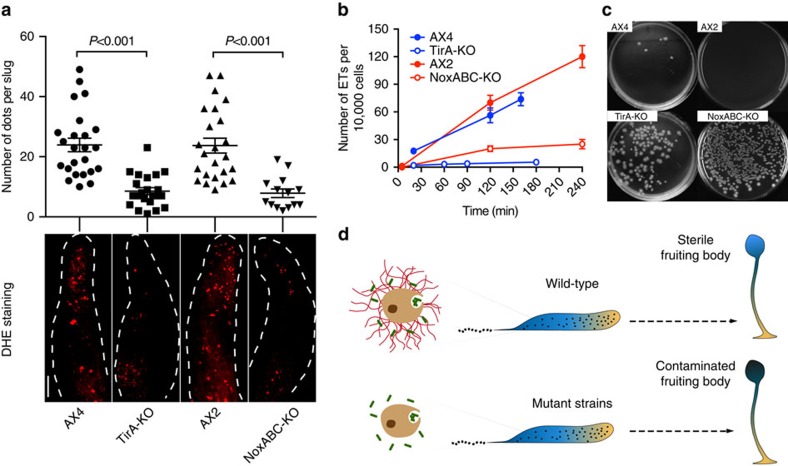
Genetic requirements for ROS and ETs formation and fruiting body sterility. (**a**) *In situ* ROS production (DHE staining, red) in wild-type (AX4, AX2) and mutant (TirA-KO, NoxABC-KO) slugs was visualized by confocal microscopy and quantified after 3D reconstruction by counting the number of red foci per slug, the *P* values were calculated using the student *t*-test, *n*>14. See also Supplementary Movie 6. (**b**) On LPS stimulation, ETs production from disaggregated slug cells from wild types and mutant strains were quantified by counting the extracellular DNA fibres stained by SYTO9 or PI, *n*=3. (**c**) *K.p.* c.f.u. generated from isolated fruiting bodies from wild types and mutant strains. (**d**) In wild-type slugs (upper), S cells not only kill engulfed bacteria, but also produce ETs to trap and kill extracellular bacteria, leading to the formation of sterile fruiting bodies. Deficiency of ET formation in mutant strains (lower) results in inefficient bacteria clearance and contaminated fruiting bodies. Scale bar, 100 μm.

Various Toll-like receptor pathways can stimulate Nox2 to generate ROS, thereby triggering ET formation by human neutrophils^8^,^22^. Neutrophils from CGD patients with deficient Nox2 cannot produce an oxidative burst nor undergo ETosis, which allows severe and recurrent bacterial and fungal infections^8^. In *D. discoideum*, the TirA protein contains a Toll/interleukin receptor (TIR) domain and is important for bacterial killing by S cells^15^. In addition, three *D. discoideum* enzymes (NoxA, NoxB and NoxC) are homologous to human Nox2 (refs 23, 24). Thus, from an evolutionary point of view, it was crucial to test their potential role in the formation of ETs. We observed that the development cycle of the TirA single KO strain and a newly generated NoxA, B and C triple KO strain was not inhibited. Both strains are able to produce migrating slugs, S cells and finally culminate into fruiting bodies (Supplementary Figs 4 and 5). The *in situ* ROS assay performed on slugs developed from *K.p.* bacterial lawns revealed that the TirA-KO and the NoxABC-KO slugs generated significantly less ROS-positive foci than their parent strains, AX4 and AX2, respectively (Fig. 5a and Supplementary Movie 6). In addition, the number of ETs produced by LPS-stimulated slug cells from TirA-KO and NoxABC-KO strains was significantly lower compared with wild types (Fig. 5b).

The migrating slug is an intermediate form before the amoebae produces a spore-holding fruiting body. Various cell-autonomous mechanisms contribute to clearing of an infection before and during the aggregation stage (Lopez-Jimenez, Soldati and Hagedorn, unpublished data), but S cells in the slug are likely a major defence to ensure production of a sterile spore population and subsequent progeny. Therefore, defects in ET formation might lead to difficulties in clearing the infection during the last stages of development. To test this, cells of the TirA-KO, NoxABC-KO and their wild-type parental strains were plated and grown on a lawn of *K.p.* until nutrient depletion, which induced aggregation and formation of fruiting bodies. We randomly picked 15 fruiting bodies from each strain, disaggregated and plated them on nutrient agar overnight at 37 °C to allow the growth of bacteria while preventing spore germination. The difference between the KO strains and their wild-type parents were striking (Fig. 5c). These data strongly indicate that, during completion of the developmental cycle, ROS-induced ET generation by S cells contribute to the clearing of bacteria in the slugs, leading to the generation of quasi-sterile fruiting bodies (Fig. 5d). However, since TirA and NOX genes might play multiple roles in the regulation of the innate defence, we do not exclude that other unknown defects due to the ablation of these genes might also contribute to the production of contaminated spores.

## Discussion

Our last common ancestor with amoebazoa dates back about 1 billion years ago^25^. During this extended period of time, the transition from unicellular to multicellular organisms took place^26^, and this was accompanied by the increasing challenge of eliminating invaders in a cooperative and/or altruistic manner to protect the integrity of the ‘community'. In this context, converting the constitutive feeding machinery of early free-living eukaryotes into a functional innate immune system appears as the most efficient strategy^13^. In addition, using simple oxygen-containing radicals as signalling molecules allowed the evolving organisms to make rapid responses to environmental changes^27^ and respond to invasion. The genetically tractable amoeba *D. discoideum* provides an exceptional model to study the roles of conserved genes involved in the evolution of multicellularity and innate immunity. Our current observations suggest a fundamental and functional conservation between S cells and neutrophils, including the use of TIR domain-containing proteins as signal transducers for the LPS stimulus, the use of NOXs for ROS generation and the common use of ROS as signalling molecules to trigger ET generation. This is the first report demonstrating that DNA-based cell-intrinsic defence strategies evolved about 1 billion years ago, long before the emergence of metazoans.

Indeed, until now, only a few reports have shown that ETs can be formed by immune cells of primitive animals, including invertebrates^10^. Amazingly, using extrusion of DNA as a weapon to trap and kill microbes that cannot be phagocytosed has its rationale^5^. During the emergence of multicellularity, a primitive innate immune system developed in the form of a dedicated set of specialized phagocytic cells, for example, the S cells that patrol the slug. This professionalization of immunity later allowed the evolution of sophisticated defense mechanisms including the sacrifice of a small set of phagocytes such as neutrophils by NETosis. Therefore, we postulate that this altruistic behaviour emerged in steps, starting from the release of ‘dispensable' mtDNA, as performed by *D. discoideum* S cells. One might anticipate that in the near future, more examples of the rise and fine-tuning of ETs during evolution of early metazoans will emerge. Consequently, the revelation of the long evolutionary history of DNA-based ETs as a conserved weapon of innate immune defence may transform our understanding of anti-microbial defence mechanisms^28^,^29^, immunodeficiency, as well as inflammatory and autoimmune conditions^30^,^31^,^32^.

## Methods

### *D. discoideum* and bacteria culture

The *D. discoideum* laboratory strains AX2 (Ax2–214) and AX4 (Ax4(Ku)^33^ have been deposited at and can be obtained from the Dicty Stock Center (http://dictybase.org/StockCenter/StockCenter.html), the derived mutant strains AX2 NoxABC-KO and AX4 TirA-KO were generated and are kept in the laboratories of TS and AK, respectively. All *D. discoideum* strains were cultured axenically in HL-5 medium (Formedium) supplemented with 50 U ml^−1^ penicillin and 50 μg ml^−1^ streptomycin (Pen/Strep) at 22 °C in 10 cm Petri dishes. The exponentially growing cells at about 80% confluence were harvested for experiments. To obtain higher cell numbers, *D. discoideum* was transferred into shaking flasks with HL-5 medium (plus Pen/Strep) at 22 °C, and cells were harvested before the density reached 5 × 10^6^ per ml (ref. 34). The avirulent laboratory bacterial strain of *K.p.*^35^ was cultured in SM medium without the antibiotic. The Kp–GFP was kindly provided by Dr P. Cosson (University of Geneva), and cultured at 37 °C in SM medium with 100 μg ml^−1^ of ampicillin.

### Development of *D. discoideum* slugs

As the experiment requires, *D. discoideum* slugs can be developed from axenic culture or from a *K.p.* bacterial lawn. To remove the nutrient medium, *D. discoideum* axenic cultures were centrifuged twice at low speed and re-suspended in Sorensen buffer. The cell pellet was then plated in a single line on a 1% water agar plate, with dyes (PI or Lucifer Yellow) added while pouring the plate^15^. Alternatively, *K.p.* cultured from SM medium were spread on SM agar plates to generate a *K.p.* lawn after overnight culture at 37 °C. *D. discoideum* cells were then added on the *K.p.* bacteria lawns and incubated at 22 °C until phagocytic plaques appeared. The actively replicating cells at the rim of plaques were gently collected with a sterile toothpick and spotted on 1% water agar plates. To increase the efficiency of slug formation, multiple inocula can be spotted on each plate^36^. The plates for both methods were mildly dried under a hood for 5–10 min and covered with aluminium folio leaving a 2-mm hole on the side opposite the lines of cells to generate a unidirectional light source. Finally, the plates were placed in a humid box at 22 °C with constant lighting. The migrating slugs usually appear 18 h post incubation and remain as long as 48 h. Of note, it is easier to generate slugs from AX2 and strains derived thereof, using the ‘bacteria-toothpick' method than the axenic development method.

### Disaggregation of slugs

Slugs that migrated more than 1 cm from the original spot were carefully collected with a pipette tip and transferred into a 1.5 ml Eppendorf tube containing 1 ml of Sorensen buffer supplemented with 1 mM EDTA^37^. Up to 15 or 20 slugs can be transferred into 1 tube to increase the cell density. The slugs were incubated in buffer for about 10 min, and then disaggregated by gentle shaking. The disaggregated slug cells were pelleted by quick spin and re-suspended in Sorensen buffer for later use.

### FACS sorting of S cells

Slugs were developed on agar plate containing Ethidium Bromide (EB) or other fluid phase dyes, and disaggregated as described above. Then, the S cells stained by EB or other fluid phase dyes, were purified by fluorescence-activated cell sorting (BD FACSAria), as described previously^15^. The top 1% fluorescent cells (S cells) and the rest of the cell population were collected separately in tubes containing ice-cold Sorensen buffer for later use. The purified S and non-S cells were then exposed to *K.p.* bacteria or 5 μg ml^−1^ of *K.p.* LPS for 2 h followed by PI or SYTO9 staining to visualize DNA.

### Cell viability and mtDNA analysis

S cells and non-S cells were purified and exposed for 2 h to 5 μg ml^−1^ of *K.p.* LPS, or the combination of LPS and *K.p.* (multiplicity of infection=50:1). Two hundred cells from each group were then plated on *K.p.* lawns. The number of viable amoebae cells was determined by counting plaques formed on *K.p.* lawns after 7 days.

Purified S cells were exposed to 5 μg ml^−1^ of *K.p.* LPS for 3 h, and then the medium and cells from culture plates were collected into 1.5 ml centrifuge tubes. The supernatant and the cells were separated by centrifugation (5 min at 1,200*g*), and the cell pellet was reserved for DNA preparation. Additional centrifugation (5 min, 4,500*g*) was applied to remove all debris and floating cells from the supernatant. The final supernatant contained mainly ETs. This was confirmed by staining an aliquot with SYBR Green and examining it by phase contrast and fluorescence microscopy. DNA was prepared by the HotSHOT method^38^ from the final supernatant fraction, and from untreated S cells as a control. The quantitative PCR was performed as described^15^ using primers (listed in Supplementary Table 1) targeting the mtDNA-encoded *rnlA* gene, a nuclear-encoded gene histone H3a and any of the 40 nuclear-encoded actin genes. The fold enrichment of mtDNA versus nuclear DNA in the LSP-stimulated S cell pellet and the supernatant fraction were calculated using the ΔΔCt method, by comparison to the non-stimulated S cell pellet as a control. The s.d. of three replicates were calculated and displayed following the Applied Biosystems' guideline for Real Time Quantitative PCR as the range of variation.

### Visualization of ETs

ET visualization can be performed on either disaggregated slug cells or FACS-purified S cells. In disaggregated slug cells, 2.5–20 μg ml^−1^ of LPS from *Klebsiella pneumoniae* (Cat: L1519, Sigma) or *Pseudomonas aeruginosa (P.a.)* (Cat: L9143, Sigma) was added to stimulate ETs formation. About 0.5 μg ml^−1^ of PI or 5 μM of SYTO9 was added to visualize ETs. Green fluorescent latex beads (Cat:15702, Polysciences) or Kp–GFP (washed twice in Sorensen prior to use) can be added before observation by time-lapse microscopy (ImageXpress Micro XL, Molecular Devices) or standard epifluorescence microscopy (Axiovert 135, Zeiss). Videos were recorded using white light, FITC and DsRed filters with a minimum exposure time, every 5 min for at least 4 h with temperature control at 22 °C.

Alternatively, to directly visualize the fate of mtDNA during ET generation, vegetatively growing cells were incubated with 5 μM MitoSox Red (a mitotropic DNA dye, Cat: M36008, Life Technologies) for 30 min. After removal of excess dye, vegetative cells were developed into slugs. Finally, the slugs were disaggregated and incubated with *K.p.* LPS for 2 h in dishes before ET imaging. To visualize the fate of mitochondrial proteins from S cells, disaggregated slug cells were incubated with 0.1 μM MitoTracker Green (a mitotropic thiol cross linker dye Cat: M-7514, Life Technologies) for 30 min. After removal of excess dye, the disaggregated slug cells were incubated for an additional 2 h with *K.p.* LPS in dishes before ET imaging.

### Measurement of bacteria viability

The live and dead *K.p.* bacteria were visualized by using the BacLight bacterial viability assay kit (Invitrogen). All bacteria are stained by SYTO9 and dead bacteria by PI. S cells from AX4 slugs were purified and mixed with *K.p.* at a 5:1 ratio of bacteria to amoebae to stimulate ET production. The live and dead bacteria in the floating suspension or attached to the ETs were visualized by SYTO9 and PI, respectively. The bacteria were judged to be associated with ETs if their fluorescent signal overlapped the fluorescence from the ET DNA stain. Bacteria not associated with ETs were judged to be in floating suspension, but we could not exclude the possibility that they were associated with an ET fragment, or that they had been associated with an ET at some previous time. In microplate reader, the viability of Kp–GFP was measured by fluorescence intensity of the GFP signal at 400 nm excitation and 512 nm emission.

### Visualization of S cells and ROS

S cells were visualized in confocal microscopy (LSM700, Zeiss) by spraying 5 μg ml^−1^ AO (Cat: A1301, Life Technologies) on slugs or by allowing slugs to migrate on water agar plates containing 2 μg ml^−1^ Lucifer Yellow (Cat: L0259, Sigma). *In situ* superoxide production in slugs was visualized by spraying 30 μM DHE (Cat: 37291, Sigma) on slugs with 30-min incubation in the dark before observation by confocal microscopy. Of note, to avoid auto-oxidation, the DHE working solution is always freshly prepared before each experiment, and the DHE stock solution is kept in the dark at −20 °C for <10 days after dissolving the powder. Usually, a × 5 or × 10 magnification fits the slug scale with optimal resolution. The object-based 3D reconstruction from stacks of confocal sections and the spatial analysis of pixel intensity were performed with the Imaris software. The results are plotted as 2D histograms of the Lucifer Yellow and DHE channels, allowing to calculate a Pearson's coefficient for the degree of colocalization.

To compare the number and location of foci of *in situ* ROS production in slugs from different strains, DHE was gently sprayed on slugs and imaged as described above. The 3D reconstruction of each confocal stack was automatically performed by the ImageJ software (see Supplementary Movie 6). Because of the three-dimensionality of the data set, it turned out that the easiest and optimal method was to rely on blind counting by three different lab members of the number of red foci in each slug, while rotating the 3D objects back and forth.

OBG-coated beads were generated to visualize phagosomal ROS generation from S cells. As described^20^, OBG (Cat: O-13291, Life Technologies) and Alexa fluor 594 (Cat:A20004, Life Technologies) were covalently linked to BSA-coated 3 μm carboxylated silica beads (PSi-3.0COOH, Kisker Biotech), and fed to disaggregated slug cells. During phagocytosis, the ROS-insensitive dye Alexa fluor 594 exhibits stable red fluorescence, while the ROS-sensitive dye OBG changes to green fluorescence due to the oxidation by phagosomal ROS, therefore giving the beads a yellow colour. The videos were recorded as described above.

### Quantification of ROS and ETs

Extracellular H_2_O_2_ production from disaggregated slug cells was quantified by membrane-impermeant AUR as described^20^. Disaggregated slug cells suspended in Sorensen buffer were added to a 96-well plate (Cat: 236108, Nunc) at 2 × 10^5^ cells per well, followed by addition of AUR and HRP (Cat: 10108090001, Roche) to the final concentration of 6.25 μM and 0.005 U ml^−1^, respectively. The volume of each well was brought to 100 μl with Sorensen buffer. About 20 μg ml^−1^ of *P.a.* LPS and/or various concentrations of catalase (Cat: C9322, Sigma), as indicated, were added to selected wells before detection. The H_2_O_2_-oxidized AUR was specifically recorded at 530 nm excitation and 590 nm emission from each well, every 2 min for 1.5 h in a microplate reader (Synergy Mx, BioTek) with temperature control at 22 °C. The slopes (relative fluorescence units (r.f.u.) per min) of each fluorescence curve were calculated as the representation of their extracellular H_2_O_2_ generation rate. At least three independent experiments were performed for statistical analysis.

ETs from disaggregated slug cells were quantified by the membrane-impermeant DNA dye PI (Cat: P4170, Sigma) in a microplate reader, at the final concentration of 0.5 μg ml^−1^. The intensity of PI-stained DNA was detected at 300 nm excitation and 590 nm emission. Similarly, the fluorescence intensity of Kp–GFP was detected at 400 nm excitation and 512 nm emission. DNase I was added to a final concentration of 100 U ml^−1^ for DNA digestion. Fluorescence signals of 13 different positions, covering 80% of the bottom area of each well were recorded and averaged every 5 to 20 min, depending on the sample size, for at least 4 h. At least three independent experiments were performed for statistical analysis.

Quantification of ETs from wild types and mutant strains was performed on disaggregated slug cells. About 5 μg ml^−1^ of *K.p.* or *P.a.* LPS was added to disaggregated AX4 and TirA-KO or AX2 and NoxABC-KO slug cells together with PI (0.5 μg ml^−1^) or SYTO9 (5 μM). The fluorescent DNA fibres (ETs) and cell numbers were counted at the indicated time points. The results were normalized as the number of ETs per 10,000 cells from more than 3 independent experiments.

### Generation of KO strains

The NoxA, B and C triple KO strains were generated in the AX2 parent strain by the Cre-Lox technique (Supplementary Fig. 3). The construction of knockout vectors for NoxA, NoxB and NoxC shared the same strategy. The Blasticidin resistance (Bsr) cassettes from the original vectors described (generous gift of Dr B. Lardy (CNRS, France)^23^), were exchanged for the ‘LoxP-Bsr-LoxP' cassette released from pLPBLP plasmid^39^. The pLox-NoxA vector was electroporated into AX2 cells, followed by PCR selection to obtain independent single NoxA-KO clones carrying the floxed Bsr cassette. To obtain double or triple KO clones using the same selection marker, the Bsr cassette was removed by transfecting the Cre recombinase expression plasmid pTX-NLS-Cre, leaving a LoxP site and three serial stop codons in both directions. This process was further repeated with the transfection of pLox-NoxB and then pLox-NoxC to obtain the NoxAB double KO and finally the NoxABC triple KO strains, respectively. The various KO clones were confirmed by genomic DNA PCR, using the primer pairs listed in Supplementary Table 1. Primers were designed to target the genomic DNA flanking each Nox gene sequence, so that the length of the PCR products can be used to confirm the successful clones due to the insertion and deletion of the Bsr cassette inside of each Nox gene.

## Additional information

**How to cite this article:** Zhang, X. *et al*. Social amoebae trap and kill bacteria by casting DNA nets. *Nat. Commun.* 7:10938 doi: 10.1038/ncomms10938 (2016).

## Supplementary Material

Supplementary Information Supplementary Figures 1-5 and Supplementary Table 1

Supplementary Movie 1 ET formation from disaggregated slug cells.

Supplementary Movie 2 ETs trapping latex beads.

Supplementary Movie 3 ETs killing extracellular bacteria

Supplementary Movie 4 3D reconstruction of in situ colocalization of S cells and ROS.

Supplementary Movie 5 ROS generation from S cells during phagocytosis.

Supplementary Movie 6 3D reconstruction of in situ ROS signals from slugs of wild type and KO strains.
