# Supplementary material for: Social amoebae trap and kill bacteria by casting DNA nets
Source: Nat Commun. 2016 Mar 1;7:10938. doi: 10.1038/ncomms10938 (PMC4773522; doi:10.1038/ncomms10938)
Supplement: Supplementary Information — Supplementary Figures 1-5 and Supplementary Table 1 [file ncomms10938-s1.pdf]

Supplementary Information

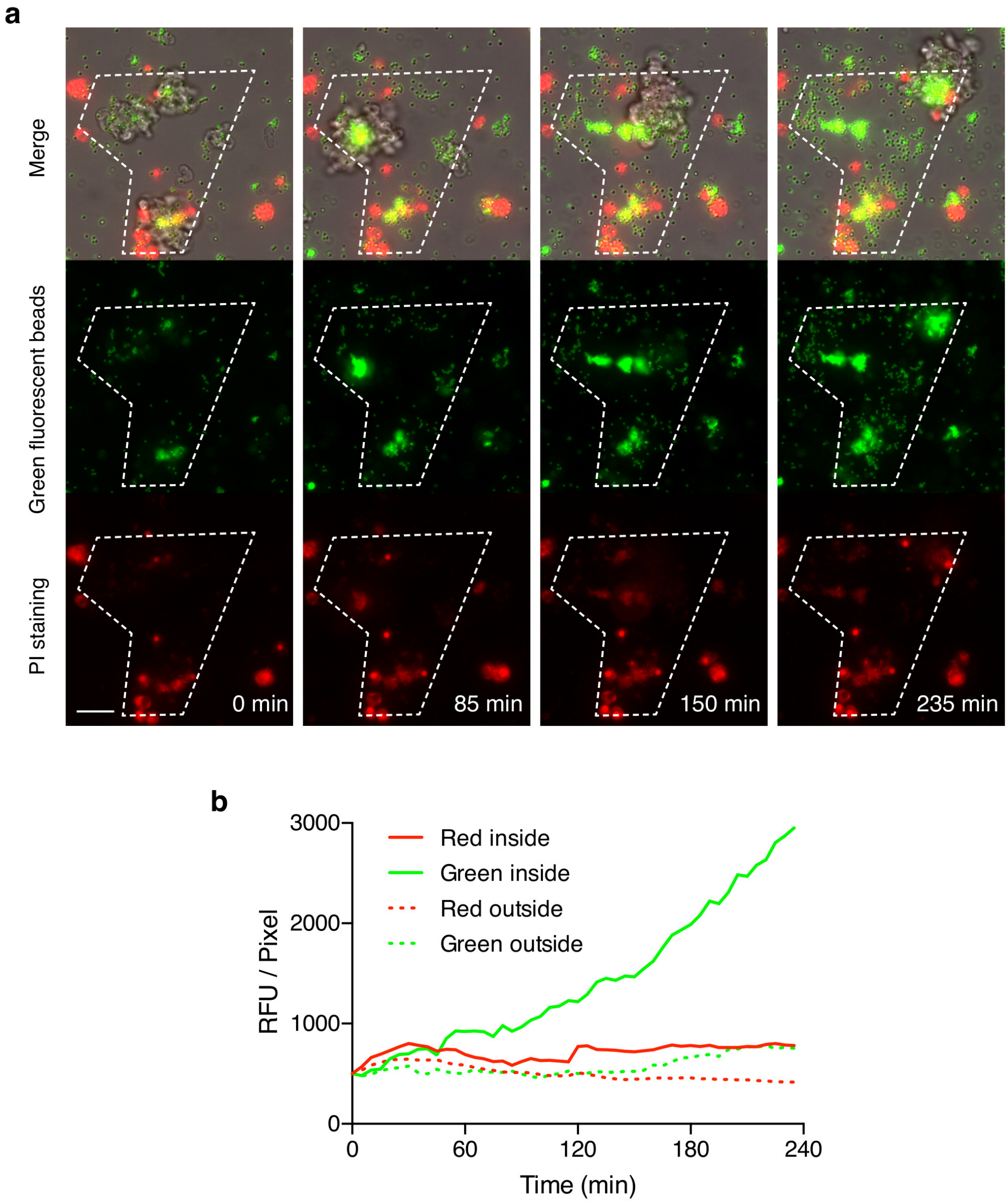

Supplementary Figure 1 | ETs trap extracellular particles. (**a**) Image gallery of Supplementary Movie 2. The disaggregated slug cells were incubated with LPS, 1 µm green fluorescent latex beads and PI. The dashed line outlines the migration zone being explored by cells and clusters of disaggregated slugs during the time of the experiment. The green and red channels correspond to the latex beads and PI-stained extracellular DNA, respectively. (**b**) Quantification of the fluorescence intensity of green and red channel inside and outside the outlined region over time for Supplementary Movie 2, as a representative result from more than 5 similar movies.

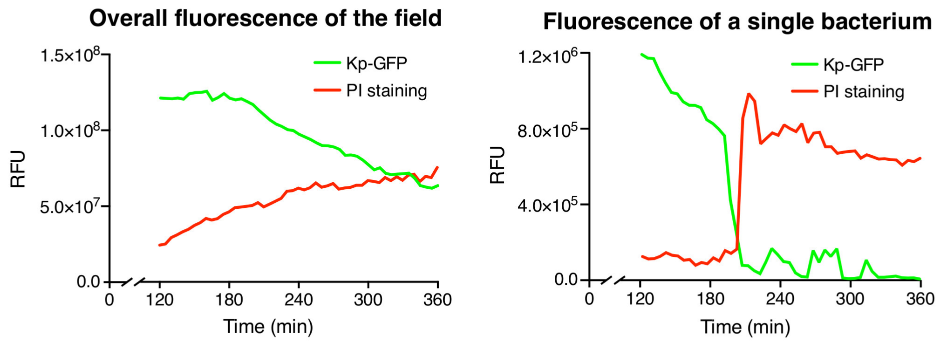

Supplementary Figure 2 | ETs kill extracellular bacteria. The overall quantification of Supplementary Movie 3 shows a general decrease of green fluorescence (Kp-GFP) and increase of red fluorescence (PI staining both ETs and dead bacteria) over time. A single bacterium, marked by an arrowhead in the movie, exhibits dramatic GFP-fluorescence loss and PI-fluorescence gain at around 200-min as it dies. This quantification for Supplementary Movie 3 is a representative result from more than 4 similar movies.

a

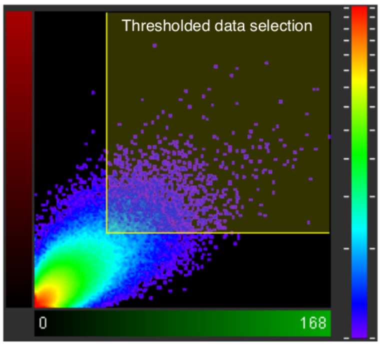

b

Parameter	mean ± s.e.m.
Pearson's coefficient in dataset volume	0.80 ± 0.01
Pearson's coefficient in ROI volume	0.52 ± 0.03
Pearson's coefficient in colocalized volume	0.48 ± 0.03

Supplementary Figure 3 | *in situ* colocalization of S cells and ROS. AX4 slugs developed on *K.p.* bacteria lawn were sprayed with DHE and AO, and 8 independent slugs were imaged in confocal microscopy as described in the methods section, n=8. (**a**) A representative 2D histogram of the green and red channels was shown. Regions of interest (ROI) and thresholds were manually adjusted, and pixel intensity spatial correlation analyses were automatically performed on each of the samples using the Imaris software. (**b**) The summary of resulting Pearson's coefficients is listed, indicating a significant correlation between AO staining (S cells) and DHE staining (ROS). Correlation coefficient: 1 = total positive correlation, 0 = no correlation, -1 = total negative correlation.

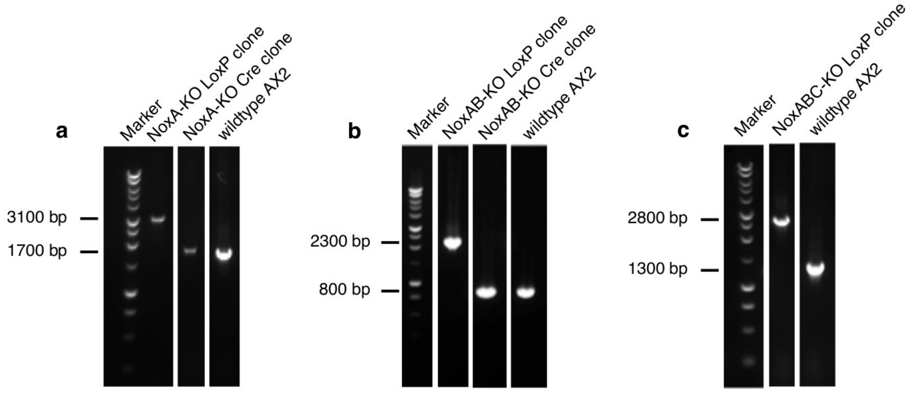

Supplementary Figure 4 | Generation of the triple NoxABC-KO strain. (**a**) The NoxA single KO strain was generated by transfecting pLoxP-NoxA plasmid into AX2 wild type cells, followed by clone isolation on a *K.p.* lawn. Insertion of the Bsr cassette increased the size of the NoxA PCR product. The NoxA single knockout (NoxA-KO) was then transfected with pTX-NLS-Cre plasmid to excise the Bsr cassette, resulting in a PCR product differring from the one in wild type AX2 only by the remaining LoxP site. (**b**) The NoxA-KO without the Bsr cassette was used to generate the NoxA-NoxB double knockout (NoxAB-KO) following the same strategy by transfecting pLoxP-NoxB and pTX-NLS-Cre plasmids sequentially. The size shift of NoxB PCR products was used to identify positive clones. (**c**) The NoxAB-KO double knockout without Bsr cassette was transfected with pLoxP-NoxC to generate NoxA, NoxB and NoxC triple knockout (NoxABC-KO). The clones were confirmed by the increased size of the NoxC PCR product due to Bsr cassette insertion.

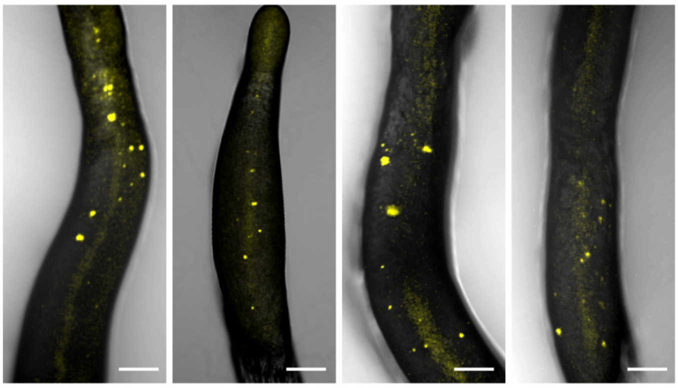

Supplementary Figure 5 | S cells visualization in slugs from wild type and mutant strains. Slugs were developed on agar plates containing Lucifer Yellow, and the S cells were visualized *in situ* by confocal microscopy. Representative examples show that TirA-KO and NoxABC-KO produce S cells. However, the fluorescence intensity from TirA-KO and NoxABC-KO S cells is less than that from wild types, indicating somehow compromised S cell functionality. Scale bar: 100 µm

Primer	Sequence
rnl-F	TGATCCAATAGTTCTGTGTGGA
rnl-R	CCGAACCACATAACAGATATGA
H3a-F	GGTTCTAAACAAGCCCATAAACA
H3a-R	CTCTAAGAGCGACAGTAC
act-F	TGATATGGAAAAAATCTGG
act-R	GCTTTTGGATTTAATGG
NoxA-F	AATAGATTATTATAATAGTCCAGCATTTGAAG
NoxA-R	CGGTAGTTCTAAGTTTTCAAAGTTTTC
NoxB-F	ATCAATGTGGAGAATTAGAAGACC
NoxB-R	TGTAAATTTGTAATGTGACATTTTTGAGC
NoxC-F	AATTTGCAAATCCATTGTCTTTTCC
NoxC-R	GGTTATTTTTTGTTCTTCACTACCAAC

Supplementary Table 1 | Primers used in this study
